# The Profiles of Body Image Associate With Changes in Depression Among Participants in Dance Movement Therapy Group

**DOI:** 10.3389/fpsyg.2020.564788

**Published:** 2020-10-06

**Authors:** Päivi Pylvänäinen, Katriina Hyvönen, Joona Muotka

**Affiliations:** ^1^Tampere Psychiatric Unit, Tampere City Mental Health Services, Tampere, Finland; ^2^Faculty of Social Sciences/Psychology, Tampere University, Tampere, Finland; ^3^Department of Psychology, University of Jyväskylä, Jyväskylä, Finland

**Keywords:** dance movement therapy, depression, body image, group therapy, treatment outcome, attachment style, mindfulness skills, women

## Abstract

This mixed-methods study analyzed the body image quality of 143 patients with depression. The participants received a 20 × 75 min dance movement therapy (DMT) group treatment, sessions twice a week. Body Image Assessment (BIA) was the data collection tool, with pre-, post-, and 3-mos follow-up assessments. Pre-intervention body image quality characteristics were low energy and activity levels, discomfort, shame and disgust toward the body, tension in social interactions. On the BIA scores, a statistical method of Latent Profile Analysis was utilized to identify participant profiles in the data. The two identified profiles were participant with initial negative body image and participant with initial neutral body image. Depression symptoms were measured with BDI, and symptoms decreased for both participant profiles following the DMT intervention. The neutral profile participants had a significantly lower depression level, better energy level, and more frequently used mindfulness factors of acting aware, non-judgmentality and non-reactivity (measured with FFMQ). Findings suggest a systemic interaction between depression symptoms, body image, attachment style, activity level, and mindfulness skills. In an interactive DMT setting it is possible to address all of these factors simultaneously.

## Introduction

Depression is considered a mental health condition, but many of its symptoms effect the body. ICD-10 (WHO, 2016) identifies depression symptoms as lowered mood, reduced energy, and decreased activity. These are physical phenomena as well. Patients show a reduced capacity for enjoyment, interest, and concentration. Often there is marked tiredness after even minimal effort. Sleep disturbances are frequent, and appetite diminished. Self-esteem and self-confidence are low, and feelings of guilt or worthlessness are often present (Michalak et al., 2019). Patients frequently describe anhedonia, and sometimes loss of libido and psychomotor retardation. Some may also experience agitation and irritability. These symptoms in their physicality are all linked to the way an individual experiences her body, both physically and emotionally. It is surprising, therefore, how little we know about how a depressed patient experiences her body.

To our knowledge, there has been eight papers (Stewart et al., 1994; Jeong et al., 2005; Koch et al., 2007; Röhricht et al., 2013; Papadopoulos and Röhricht, 2014; Punkanen et al., 2014; Pylvänäinen et al., 2015; Pylvänäinen and Lappalainen, 2018) that have studied depression in the context of embodied experience. All these papers have studied the use of dance movement therapy (DMT), or body psychotherapy facilitated by a dance therapist, in the treatment of depression.

### Dance Movement Therapy and the Treatment of Depression

Embodiment is a perspective that recognizes human embeddedness in a sensing, responding, and active body (Aphosyan, 1999; Tschacher and Bergomi, 2011; Ziemke, 2016). For a dance movement therapist, embodiment, i.e., connectedness to one’s body and attentiveness to the sensory information the body offers, is a natural area to attend to and investigate. DMT, which is classified as a creative arts therapy, is defined as the therapeutic use of movement to further the emotional, cognitive, physical, spiritual, and social integration of the individual (European Association of Dance Movement Therapy, 2020). It aims to engage patients in physical and verbal exploration of their experiences during movement-based interaction.

The use of DMT in the treatment of depression in adults or adolescents has been well documented (see, for example, Stewart et al., 1994; Jeong et al., 2005; Koch et al., 2007; Röhricht et al., 2013; Punkanen et al., 2014; Pylvänäinen et al., 2015; Pylvänäinen and Lappalainen, 2018; Hyvönen et al., 2020). DMT treatment has typically proven effective in alleviating the depression symptoms of participants. Koch et al. (2019) conducted a meta-analysis on the effects of dance and DMT interventions on depression. Based on the outcomes of 18 published studies, the mean effect size (ES) was 0.54, *p* < 0.001, which indicated a moderate significant effect.

### The Tri-Partite Model of Body Image

Body image is defined as the lived and experiential perception of the body, and the psychological significance the body has for the individual. The present study uses the tri-partite model of body image. The tri-partite model categorizes body image information into three elements: image properties, body self, and body memory (Pylvänäinen, 2003, 2012, 2018).

Image properties consist of perceptions, thoughts, judgments, and socio-cultural values related to the physical appearance of the body. Image properties frequently reflect the perspective of an external gaze or even critical observer. This observation may be perceived from others or it may be internalized, the person subjecting herself to external gaze. Image properties evoke emotional responses in the person, which are subsequently experienced through the body self.

Body self is the active, responsive aspect of the body image. Body self refers to experiences and patterns in the present being, and in interaction with the world and with others. The body self is actualized through the sensory, kinesthetic, and perceptual information in the body. This information not only relates to external surroundings, but also to the state prevalent within the individual. The responses of body self may have an affective quality.

Body memory refers to the embodied information stored in the body. A phenomenological perspective outlines body memory in three spheres: habitual, traumatic, and erotic (pleasurable) body memory (Casey, 1987). Fuchs (2012) and Koch et al. (2012, 2013) propose a more detailed categorical differentiation, which specifies aspects of body memory that hold information about the surrounding environments and incorporate social habits and embodied patterns. This is echoed in the proposal made by Pylvänäinen and Lappalainen (2018): reciprocity in interaction and attachment style are embodied (Schachner et al., 2005; Bentzen, 2015).

Reciprocal interaction with others is built upon, but also shapes, attachment patterns, i.e., the ways in which an individual has learned to relate to others (Fosha et al., 2009; Porges, 2009). Reciprocity in interaction is observed in the shaping and posturing of the body and body movements, levels of arousal, attention, and vocal expressions in response to what is perceived from another’s expressions. The essence of these patterns is stored in the body memory, while actions in the present are shaped by the body self.

Pylvänäinen and Lappalainen’s (2018) study of the body image used a sample of 18 adult patients with depression in outpatient psychiatric treatment. They completed the Body Image Assessment (BIA) before and after twelve 90-min sessions of DMT group treatment. Before treatment, the qualitative themes of low energy, lack of direction in action, and lack in the sense of agency in social situations were typical contents in the body image. Participants experienced shallow, fragmented, negative, and tense habitation in their bodies. Pains and worry about weight were frequently mentioned. After the short term DMT group treatment participants’ body images were less negative. They described a better connection with their bodies, had greater tolerance to sensation, were more settled in their bodies, and found pleasure and meaningfulness in bodily experiences. BIA responses were also scored, and the scores analyzed quantitatively. Large effect sizes were evident in the change between pre- and post-treatment assessments. In the Pylvänäinen and Lappalainen (2018) study patients’ depression symptoms were assessed with Beck’s Depression Inventory (BDI-II), Symptoms Check List (SCL-90), Hospital Anxiety and Depression Scale (HADS), and Clinical Outcomes in Routine Evaluation Outcome Measure (CORE-OM) at three measurement points: pre- and post-treatment, and at 3-month follow-up. Change for more positive body image during the treatment predicted fewer depressive symptoms at the follow-up measurement.

### The Research Questions of the Present Study

This paper uses data collected for a larger study on the use of DMT in the rehabilitation of adult patients with depression (Hyvönen et al., 2020). The aim was to study the effect of a short-term DMT group intervention on participants’ depression. The structured DMT intervention was offered in different cities in Finland by 12 dance movement therapists. The benefit of this multi-center research was that results were more likely to be due to the effects of DMT rather than the characteristics or skills of a specific therapist (Karkou et al., 2019).

The findings on the effect of DMT on participants’ depression symptoms are reported in Hyvönen et al. (2020). This present paper focuses on participants’ body image and the ways in which it changed after a DMT intervention. Research questions addressed in this paper are:

(1) Does the larger sample show similar characteristics in the pre-intervention body image of the patients with depression as was observed in the earlier study by Pylvänäinen and Lappalainen (2018): low energy level, lack of the sense of agency in social situations, shallow experience and tense habitation of their body?
(2) Does the body image change after the short-term DMT group intervention for better?
(3) Does the body image quality impact how a participant benefits from the DMT group intervention? Can different body image profiles be identified so, that the body image profile would be associated with identifiable characteristics in depression and attachment style? Pylvänäinen and Lappalainen (2018) suggested that attachment style is embodied. The hypothesis is, that the secure attachment style would associate with more positive body image quality, and the insecure attachment style with more negative body image quality.
(4) What clinical implications do these findings have?

This paper explores the embodied experiences of patients with depression through the construct of body image. In doing so, it provides a new perspective on the understanding of depression. A mixed methods approach will be used. The data is first explored qualitatively to detect the core themes in the body image of the patients with depression, then quantitatively with the statistical method of Latent Profile Analysis (LPA) to detect different body image profiles in the data. Participants with different body image profiles can also be perceived as different participant types. The body image profiles will be identified on the basis of statistical analysis of the data, but finally an integration of the qualitative analysis and quantitative results will be applied to the data in order to delve deeper into the different participant types. The description of different participant types aims to offer practical guidelines for use in clinical practice.

## Materials and Methods

### Recruitment Procedure

The board of research ethics in the Central Finland health care district approved the research (Dnro 8U/2016). The study was funded by the Social Insurance Institution of Finland between 2017 and 2019.

Participants for the study were recruited through public mental health services, which included outpatient psychiatric clinics, primary healthcare mental health services, student health services, occupational health services, and psychiatrists in private health clinics. The opportunity to participate was advertised in three newspapers, and through social media.

Persons interested in participating in the research had a screening interview by telephone with the researchers. The criteria for inclusion were that participants should be working aged, i.e., 18–64 years of age, diagnosed with depression that impaired their ability to work or study, and that they had been assessed suitable for rehabilitative psychotherapy by a psychiatrist, receiving at least 3 months of appropriate treatment. Persons with active suicidal ideation, psychotic symptoms, and/or substance abuse were excluded from the study. Interviewees who were pregnant or suffered serious chronic pain were also omitted from the study. Participants needed, as a minimum, to be sufficiently physically able to get up from a seated position on the floor without assistance. Participants received a mailed information leaflet regarding their participation in the study and were asked to sign a research agreement.

A total of 157 participants participated in the study. A randomization procedure was applied among those participants, who were in larger cities and where the number of participants was sufficient for intervention and control group in the same city. A total of 109 participants took part in randomized intervention and control groups. With control group participants, we could assess their body image prior to the DMT group intervention and observe how a period of treatment as usual effected the body image. Treatment as usual did not include any DMT nor other body oriented treatment.

As the control group participants received DMT group treatment after the treatment as usual period, their body image data during the intervention phase could be studied together with that of the intervention groups (whether randomized or not). This resulted in a total of 143 participant responses to the pre-intervention interview (54% in the DMT intervention group, 46% in DMT-delayed control group). To detect the body image themes, body image profiles, and the participant types, this paper uses the data collected from these 143 participants. The response rate of these participants to BIA at the post-intervention point was 78%, and at the 3-month follow-up 74%.

### Participants

Participants were working aged, i.e., 18–64 years old, and the mean age was 42. Incidentally, mostly women were interested to participate in the study, and 97% of participants were female. Approximately 75% of participants had had depressive periods before. 60% were using medication to treat their depression, 40% were not. The reasons the participants most frequently gave for their depression were problems in close relationships with their partner, their parents, or their children: 70% of participants named this. 50% of participants mentioned traumatization due to abuse, physical or mental violence, or a serious accident. 35% reported that deterioration of their physical condition and fitness had resulted in depression. 32% of participants mentioned a lack of creativity and self-expression as a reason for their depression.

### DMT-Group Intervention

Twelve dance movement therapists administered the interventions during this research. All dance movement therapists had had a 4-year professional specialization training to work in this profession. In addition to this, they had attended 12 days of intervention training for facilitating the intervention in the study. During the intervention process they were provided with group supervision by senior dance movement therapists. The DMT group treatment structure was based on earlier DMT studies with depressed patients (Punkanen et al., 2014; Pylvänäinen et al., 2015). An integrative approach was employed in the treatment of depression. DMT methods which were used in the intervention included dance and movement improvisations, mindfulness practices, use of props (e.g., fabrics and balls), and reflection through drawing, writing, and discussion. Group sessions consisted of orientation to the group, thematic movement exploration, and closure. The 20 sessions were structured to cover the following themes during the 10-week process: group goals, movement options and boundaries, body awareness and resources, symbols, safety and regulation, expressing emotions, body narrative, playfulness, needs, being and doing, agency, and processing through movement the time span of the group and of the participants’ own life. In earlier studies (Punkanen et al., 2014; Pylvänäinen et al., 2015; Pylvänäinen and Lappalainen, 2018) these themes had been observed to be relevant to this patient population. These themes provided a structural backbone for creative responses from the therapist to the process the groups were working through.

### Measurement Tools

Body Image Assessment is a verbal assessment tool based on the tri-partite model of body image (Pylvänäinen, 2018). BIA captures information about the participant’s body image, i.e., of her image properties, body self and body memory. BIA can be applied as an interview, taking approximately 45–60 min, or in a questionnaire, to which the participant can reply in writing. BIA does not assume any pathology or problems in the respondent’s body image. The BIA response effectively communicates, how an individual relates to her own body and how she experiences interaction with others in the body.

Body Image Assessment can be analyzed qualitatively and quantitatively. In quantitative analysis, a three-point scoring system is used: 0 indicates negative content in response to a question, 1 indicates a neutral response or the response equally identifies pros and cons, and 2 indicates positive content. The negativity – positivity of the content is the focus of scoring. Negative content expresses avoidance, discontent, discomfort, lack of physical agency, or negative judgment. Positive content expresses sensing, acceptance, good physical agency, comfort, or pleasure. The sum of the four questions can range from 0 to 8. Scoring is applied to the first four BIA questions, which do not address the participant’s opinion of what is important in their body in general, nor their body memory, since positivity and negativity are fundamental and inherent qualities in memories of pleasure or suffering.

The Relationship Questionnaire (RQ) (Bartholomew and Horowitz, 1991) is a tool for measuring adult attachment styles. Attachment style refers to the person’s internalized patterns of settling and being, and behavior in close relationships. Attachment can be secure or insecure. Insecure attachment can be characterized as fearful/disorganized, preoccupied, or avoidant/dismissive. In the first part of the questionnaire the respondent evaluates which of the four alternative attachment patterns corresponds best to her general relationship style. In the second part the respondent rates, on a 7-point Likert-type scale, the degree to which each of the attachment patterns resemble her own patterns. This questionnaire captures the participant’s own general impression of her attachment style.

The Five Facet Mindfulness Questionnaire (FFMQ) (Baer et al., 2006) assesses mindfulness skills through five factors: observing, describing, acting aware, non-judgmentality and non-reactivity. The questionnaire consists of 39 statements, which the respondent evaluates on a 5-point Likert-type scale. This questionnaire is used as a tool for assessing a participant’s style of relating to her experiences. The FFMQ was used for assessing how mindfulness skills related to a participant’s body image.

Activity level was assessed with a single item from the Work Ability Index (WAI) Psychological Resources section: “Have you recently been active and alert?” (Rautio and Michelsen, 2014). The participant responds to the question on a scale from always (1) to never (5). The lower the score, the more frequently the participant has felt active and alert. In this study this item was used to capture the participant’s perception of her activity level.

Beck’s Depression Inventory (BDI) (Beck et al., 1961, 1988, 1996) measures depression symptoms with 21 items. It is frequently used in clinical assessment to assist recognition of depression and its severity, and in research as a means of measuring treatment outcomes. The higher the sum score, ranging from 0 to 63, the more severe the depression. This questionnaire was the main tool for assessing the severity of participants’ depression symptoms.

### Qualitative Analysis of Body Image Assessment Text Data

BIA interviews and written responses produced narratives, all in verbal text form. The narratives described, how an individual experienced certain aspects of her body and interacting with the body. As we received the narratives from patients with depression, they also contained lived experiences that resonate the depressive condition. The contents of these narratives were of interest in the study, as there is very little systematic knowledge about how patients with depression experience their body and embodiment. Studying narratives is one approach in qualitative research (Seale et al., 2004).

ATLAS. ti software was used to contain the qualitative analysis of the BIA responses. The contents of each respondent’s replies at pre-measurement, post-intervention measurement and at the 3-months follow-up were analyzed. For control group participants, initial BIA responses from the pre-intervention waiting/treatment as usual – time were also available. Thematic codes for the analysis were initially extracted from this control group data because it consisted solely of participants’ responses with no influence on the responses from an interviewing therapist. As these written responses were slightly shorter than the interview responses, central themes appeared more concisely in this data than in the longer interview responses. The aim with the thematic codes was to capture the characteristics of the body image of a patient with depression. We applied the same thematic codes to the data at each measurement point. To minimize bias and unintentional selection in the analysis, a rule was applied that every expression in every response must be coded into some thematic code. ATLAS. ti held the listing of the thematic codes themselves, counted the number of codings in each theme, and generated lists of the exact text quotes, which also allowed exploration of the qualitative changes within a thematic code over the course of treatment. The qualitative analysis provided a view on the contents of the body image of the patient with depression. The scoring of the BIA questions functioned as a separate analysis: it was a parallel way to assess the quality and changes of the responses.

### Quantitative Analysis: Latent Profile Analysis (LPA) for Detecting the Body Image Profiles of the Participants

Using the statistical method of LPA it is possible to statistically explore whether there are subgroups of participants within the whole sample. In this study, the aim was to determine whether there were subgroups who exhibited specific patterns of change in response to the DMT intervention. In particular we were interested in seeing, whether participants differed in the body image change patterns and if this was related to attachment style. Participants’ BIA scores at the different measurement points were used as the variable for LPA.

In the LPA, the model of best fit for the analysis is established according to several statistical fit indices (Jung and Wickrama, 2008). First, the Bayesian Information Criterion (BIC), the Vuong-Lo-Mendell-Rubin (VLMR) test, the Lo-Mendell-Rubin test (LMR), and the Bootstrapped Likelihood Ratio Test (BLRT) are calculated. The lower BIC values are, the more suitable a potential profile model is. In the VLMR, LMR and BLRT, *p* < 0.05 indicates that a model with *k* profiles is better compared to a model with *k −* 1 profiles. In our study, models with 1–6 profiles were tested. Second, a good solution is indicated when there is successful convergence and, a high entropy value (range 0–1). Third, and most importantly, the identified profiles need to be meaningful in the context of the data. This LPA was done with the Mplus statistical package (Version 7.3) with full information maximum likelihood (FIML) estimation method. All the available data was used in the analyses and missing data was assumed to be Missing at Random (MAR) (Muthén and Muthén, 2012).

The statistical 3-step method (Asparouhov and Muthén, 2013) was used in analyzing the relationships of the LPA profiles with the outcome variables of BDI, activity level, and mindfulness facets. The association of the Relationship Questionnaire (RQ)-scores and the LPA profiles was analyzed with cross tabulation and χ^2^-test in SPSS (version 26.0.0.1), because RQ is a nominal variable.

The profile differences in the BDI score change between the measurement points were compared with the HLM (Hierarchical Linear Modeling) and Wald test. Within group Effect Size (Cohen’s *d*) was calculated by subtracting from the follow-up measurement mean the mean of the pre-measurement, and then dividing this difference with the pooled standard deviation of the pre- and follow-up measurements. The corrected between groups Effect Size was calculated by subtracting from the difference in group means at the follow-up measurement the difference in group means at the pre-measurement, and then dividing this difference with the pooled standard deviation of the pre-measurements. This analysis was completed with MPlus-program; all the available data was used in the analyses and missing data assumed to be Missing at Random (MAR) (Muthén and Muthén, 2012).

### Case Portraits

Bringing together the characteristics of the body image and the variables of activity level, relationship style, and BDI-score, typical case portraits were extracted from the data. The aim of this integration of qualitative and quantitative analysis was to offer clinicians a means by which to assess the potential benefit of DMT group treatment for an individual. Furthermore, these case portraits represented a means to qualitatively explore more closely the profiles that were the outcome of LPA.

## Results

### Characteristics of the Whole Sample

The pre-measurement mean BDI score of the sample was 22 and the mode 18 points. This mean score indicated moderate depression. At the post-measurement point the BDI mean was 16, indicating mild depression, and the mode 13 points. At the 3-month follow-up the BDI mean was 16, the mode 9.

At the pre-measurement 37% of participants reported that “every now and then they had been feeling alert and active.” At the post-measurement, this increased to 39% of responses, but at the 3-month follow-up decreased to 36% of responses. Participants reporting a slightly more active experience, i.e., “feeling alert and active fairly frequently” represented 15%, 27%, and 27% of the sample at the respective measurement points.

The Relationship Questionnaire (RQ) showed that the most typical attachment style in the sample was insecure: fearful/disorganized or preoccupied in style. Participants felt that it was not easy to feel close and connected to others. They wanted to make connections with others, but it was hard to trust others or to be dependent in any way. Also, there was a fear of being harmed if allowing oneself to be too close to others. The questionnaire did not discriminate between psychological or physical closeness. The clear prevalence of insecure attachment style persisted over the three measurement points.

For the FFMQ-mindfulness questionnaire the score range can be 39–195. The score range of the responses in the whole research period was 59–182 in this sample. In the total sample the mean was 121 at the pre-measurement, 125 at post-measurement and 127 in the 3-month follow-up. The scores show a tendency toward slight increase in mindfulness skills. The change between pre-measurement and 3-month follow-up was statistically significant: *t* = −3.64, df = 107, *p* < 0.001, 2-tailed *t*-test.

The mean Body Image Assessment sum score in the total sample was 2.6 at the pre-measurement, 3.4 at the post-measurement and 3.3 at the 3-month follow-up. The sum score has a range of 0–8, so the mean remained closer to the negative end of the range. The change between pre- and post-measurement was statistically significant: *t* = −3.46, df = 104, *p* < 0.001, 2-tailed *t*-test. The increase in the score indicates there was in general a slight change for better in the body image after the DMT-group intervention.

### Qualitative Results: Central Themes in the Body Image of Patients With Depression Before the DMT Group Intervention

The qualitative analysis of the BIA responses at the pre-intervention measurement showed that the main characteristic of the body image of a depression sufferer was negativity of experience. The most negative areas tended to be participants’ image properties and their experiences of interaction with others. A bit less negatively the participants experienced physical activity and their own body when alone.

Table 1 lists the themes that emerged in the data, in order of frequency. In general, they indicate a body image loaded with tensions and discomfort, a wish to avoid bodily sensations, agonies in both social situations and in situations of aloneness. When not sensing one’s body, one tends to be judgmental of it or to think of it mostly with concern as to how others will accept one’s body or think of one’s behavior. There was a tendency to prefer not to sense the body, because that was more comfortable. Participants often stated that when a situation was good they did not particularly attend to their body. These findings resonate similar themes as were observed in the Pylvänäinen and Lappalainen (2018) earlier study with a smaller sample.

**TABLE 1:** Themes in the Body Image Assessment (BIA) of patients with depression at the pre-intervention measurement, in order of frequency.

**Theme (*x*) = the number of quotes in this theme**	**Contents in the theme**
Heavy, no energy, poor activity (169)	Pain Poor capacity for activity Tired, no energy Clumsy, poor motor skills Problems in initiation Lacking physical activity Would want to do more than one does
Own body is good (153)	Likes to take physical action Perceives one’s body as good and well capable Physical activity brings connectedness with one-self Kindness, acceptance toward one’s body Perceives one’s looks beautiful Appreciates the feminine characteristics in one’s body
Relationship with own body is troublesome (105)	Disgust Shame Sense of over-weight Weight gain because of medication Weight gain because of lack of physical activity Eating disorder behaviors
Not at ease with one’s physical being (89)	The body does not feel good The body does not feel like own The body does not feel comfortable Does not identify with the body Not breathing freely Not feeling good enough with one’s body for others
Felt troubles in social situations (87)	Anxious body responses in various body areas: neck, chest, stomach, hands Discomfort Tension Being on guard Shrinking one’s body Shame which inhibits action Context/situation dependent
Thinking of what others think/rumination (76)	Concern of own behavior Concern of social roles Recognizing fear of others in social context Lacking social interaction Worry of how others think of one-self Naming the tendency to withdraw Efforts to hide true experience/feeling Sense of confusion in moments of social attention directed at one-self
Peace and ease when alone (39)	Sense of relaxedness Freedom when alone More attention to one’s body responses Enjoyment of being as one is Allowing resting and recuperation
Tension and fear in the body when alone (36)	Being alone triggers anxiety Stressful thoughts elicit anxiety in the body, when alone Restlessness Hard to relax and calm down Sleeping difficulty Experiences of the meaninglessness of life
Disconnection when alone (16)	Not connecting with the body sensations Not recognizing body sensations No interest in attending to the body Preferring not to sense anything in the body Dissociation Feeling as if the body does not exist
Disconnection when in social situations (14)	Not attending to the body sensations in social situations Cognitively not interested in body sensations Feeling nothing in the body when in interaction with others Family tradition of not expressing emotions Remaining at distance physically

There were some positive experiences in the body as well. For some participants, physical activity was a source of pleasure and gave a sense of agency. When alone, some participants felt at ease and connected with their bodies better.

As to the contents of body memory, at the pre-intervention measurement the body memories of suffering often featured episodes of negative hyperarousal, experiences of being fearful and alarmed. Body memories of domestic violence, whether in a participant’s childhood family or adult relationships, were frequent. Body memories of suffering were linked to physical illness and hospital experiences, often in relation to giving birth. Pain and being ashamed of the body were themes in body memories of suffering. Shame was typically related to a participant’s looks and how others had commented her body. Shame was sometimes connected to sexual abuse that some participants had experienced. Finally, low arousal level, lacking energy, and feeling exhausted were also mentioned in body memories of suffering.

Body memories of pleasure were most frequently around experiences of movement and physical activity. Participants had pleasurable memories of dancing, swimming, and sports. Experiences of nature, pets, and activities in nature (e.g., fishing and camping), were described. Pleasant experiences had been captured in body memories in moments of relaxation in the sauna or when having a massage. Physical closeness, intimacy and hugs, being with one’s partner, and moments and interactions with children were mentioned in pleasurable body memories. Some participants described enjoying the body’s capacity to sense and named sensory experiences they had enjoyed. Experiences of pleasure were mostly from normal and natural moments and interactions, where it had been possible to be open to the moment and feel sufficiently safe. Participants never described anything threatening related to these situations.

### Latent Profile Analysis (LPA) Results: Negative and Neutral Body Image Profiles and Their Differences

For assessing, whether the body image quality impacts the change process, the BIA scores were the base for the LPA. Table 2 shows the statistical criteria values for the models tested in LPA. Of the models that were tested, the BIC, VLMR, and LMR scores indicated that a two-profile model was the best fit. The two profiles were named as a negative body image profile and a neutral body image profile. The analysis proceeded with exploring in this data, how participants with the negative body image profile and participants with the neutral body image profile changed differently during the research period when assessed by the variables of BDI, activity level, mindfulness skills, and relationship style.

**TABLE 2:** Information criteria values for the tested Latent Profile Analysis (LPA) models.

**Number of classes**	**BIC**	***p*${}_{\text{VLMR}}^{1}$**	***p*${}_{\text{LMR}}^{2}$**	**Entropy**	**Group sizes**	**The diagonal classification probabilities for the most likely latent class membership (column) by latent class (row)**
1	1468.136	–	–	–	143	
*2*	1455.364	*<0.001*	*<0.001*	*0.658*	*81/62*	*0.922/0.858*
3	1468.136	0.3961	0.4147	0.603	44/70/29	0.697/0.909/0.742
*4*	1470.988	0.2977	0.3148	0.695	48/26/29/40	0.869/0.761/0.768/0.816
5	1482.440	0.5530	0.5648	0.739	48/20/1/37/37	0.884/0.667/0.702/0.885/0.780
6	1493.297	0.2300	0.2363	0.875	49/32/19/1/1/41	0.901/0.736/0.676/0.719/0.976/0.914

*^1^Vuong-Lo-Mendell-Rubin Likelihood Ratio Test. ^2^Lo-Mendell-Rubin Adjusted LRT Test. Italic values significant to p < 0.05 indicates that a model with k profiles is better compared to a model with k − 1 profiles.*

**FIGURE 1:**
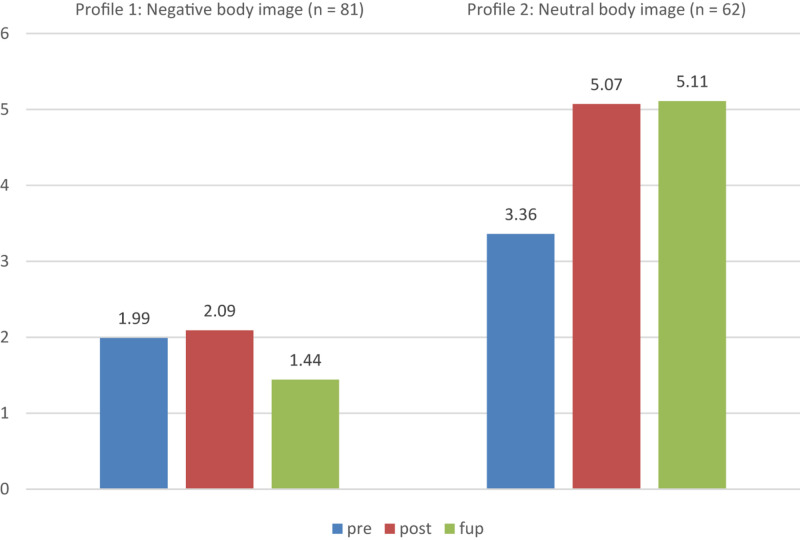
Changes in mean Body Image Assessment (BIA) scores of the two profiles generated by LPA over the three assessment points.

Table 3 shows the changes in mean BIA scores in the negative (*n* = 81; 57% of participants) and neutral (*n* = 62; 43% of participants) body image profiles over the three assessment points. The majority of participants were in the negative body image profile. After the intervention their body image remained fairly negative despite a small improvement in the scores, and this change was not lasting (see Figure 1). Participants in the neutral profile exhibited a more neutral relationship with their body at the pre-intervention, and, following the intervention, showed a greater positive change, which continued to the 3-month follow-up. These positive changes were described in the qualitative BIA responses as experiencing greater comfort in being in one’s body when alone, and, in social situations having a more accepting and reflective attitude toward one’s body and body sensations, significantly less hatred toward one’s body and body weight, and more frequently experiencing own body as good. Participants thought less about how others thought about their body.

**TABLE 3:** Latent profile analysis results: two profiles.

**Measurement point**	**Estimated means**	**Variance**
Negative body image profile (*n* = 81)		
Body image pre	1.991	2.726
Body image post	2.090	2.193
Body image 3 mos fup	1.442	1.904
Neutral body image profile (*n* = 62)		
Body image pre	3.361	2.726
Body image post	5.072	2.193
Body image 3 mos fup	5.107	1.904

When the profiles’ RQ scores were analyzed by cross-tabulation, the differences were not statistically significant [χ^2^ (df 3) = 5, *p* = 0.172]. In both profiles, the most common relationship style was insecure, and the majority of participants recognized this as characteristic of them. In the negative profile, 40% of participants reported fearful/disorganized, 39% preoccupied/contradicting, and 5% dismissive/avoidant attachment styles. In the neutral profile, the distribution was 35% for fearful/disorganized, 28% for preoccupied/contradicting and 7% dismissive/avoidant attachment styles. The only difference in relationship styles between the two profiles was that there were more participants in the neutral profile who assessed their characteristic relationship style as secure: this accounted for 30% of participants in the neutral profile, but only 15% in the negative profile.

Table 4 shows the results of a comparison between the identified participant body image profiles for differences in mean BDI, activity level, and FFMQ scores at the pre-intervention point, using the 3-step method and χ^2^-test. The two profiles were associated with significantly different mean scores in BDI, activity level, and in three factors of mindfulness, namely acting aware, non-judgmentality, and non-reactivity. The neutral body image profile exhibited a more positive systemic situation than the negative profile: they had a lower initial BDI score, greater activity level, and reported that they used the aforementioned mindfulness factors more often.

**TABLE 4:** The statistical 3-step method analysis of the relationships of the LPA profiles with the outcome variables.

	**Mean**			
**Variable**	**Negative profile – neutral profile**	**SE**	**χ^2^-test**	***p*-value**
BDI pre	25.9 16.2	1.047 1.098	34.819	<0.001
Energy level	3.70 2.90	0.119 0.107	18.960	<0.001
FFMQ pre *Total*	115.45 127.75	2.773 2.489	8.406	0.004
*Act aware*	18.89 24.92	1.018 0.695	22.815	<0.001
*Non-judge*	21.85 29.39	1.213 0.771	26.087	<0.001
*Non-react*	17.17 20.54	0.674 0.554	12.356	<0.001
*Observe*	27.66 27.20	0.728 0.851	0.132	0.717
*Describe*	25.36 25.34	0.979 1.045	0.000	0.992

*BDI, Beck Depression Inventory; FFMQ, Five Facet Mindfulness Questionnaire.*

#### Changes in Body Image and Depression Symptoms During the Research Period

The repeated measurement points employed in data collection allowed us to observe patterns of change in the participants’ body image and depression scores. As the control group participants were included in the research design, there was a period of time when these participants did not receive DMT treatment, but were receiving treatment as usual. During this period their depression scores did not change (Hyvönen et al., 2020) and their BIA scores did not change either.

We analyzed, how the depressive symptoms changed in the participant body image profiles during the research period. Table 5 shows mean BDI scores for the two body image profiles at the three measurement points during the DMT intervention phase. There were significant within-group changes in both profiles between the pre and 3 mos follow-up measurement points. Cohen’s *d* indicated medium effect size in both groups. However, there was no significant between-groups difference in how the two profiles changed over the measurement points. This finding was repeated with the Wald test, which analyzed whether the two profiles changed differently. However, in the whole sample the BDI score change between the pre and post-intervention measurement points was significant [Wald test (df = 2) = 65.70, *p* < 0.001]. On the average, the BDI score decreased 5.2 points between pre- and post-measurements.

**TABLE 5:** The BDI change patterns in the body image negative and body image neutral profiles.

**Profile**	**BDI pre Mean (sd)**	**BDI post Mean (sd)**	**BDI 3 mos follow-up Mean (sd)**	**Pre-fup Cohen *d* within group**	**Cohen *d* between group**	Wald test* df = 2
Body image negative	24.8 (7.95)	19.7 (8.93)	20.1 (8.39)	0.58	0.09	0.362 ns
(*n* = 76)						
Body image neutral (*n* = 59)	17.4 (8.19)	12.0 (8.25)	11.9 (7.87)	0.69		

*Wald test tests for the group difference in change.

In conclusion, whether a participant had a negative or neutral pre-intervention body image quality, they showed some positive change in their BDI score post-intervention. If the pre-intervention body image was less negative or more neutral, depression symptoms tended to be milder, and the outcome of the treatment was close to normal mood (BDI mean approximately 12 points). Within the data there were variations from these general tendencies, which were noteworthy even though they were not so prevalent that an LPA model with more profiles would have been a better fitting choice. The following case portrayals illuminate these variations in the data.

### Information for the Clinical Practice: Typical Cases Portrayed by the Data

The participants’ progress through DMT group treatment allowed us to trace some typical themes and trends during the process, on the basis of the qualitative analysis of BIA contents and the level of BIA scores, combined with the results of self-evaluation questionnaires, and the participant body image profiles resulting from LPA. This data offered a view into what was relevant in the participants’ present situation, what their body image was like, and how they benefited from the treatment. Exploring the data this way made it clear, that the initial BIA score was not the only determinant of the change process and treatment outcome for participants. The following four typical case types emerged:

*Difficult case* participants had fearful/disorganized or preoccupied/contradictory attachment styles. Initial activity levels were seldom or never good. There was a profound dislike of one’s body: not liking the physical appearance of the body, experiences of being called ugly, or not respected by others. Physical activity could be a strain, and this participant was easily exhausted. There could be indications of eating disorder behaviors. Participants did not typically sense their bodies, or, if they did, it was sensed in agony and discomfort. They did not understand their body, nor have a sense of connection to the body, but, rather, a sense of dissociation from it. There were frequent memories of traumatizing events, such as sexual harassment, physical violence, verbal abuse, typically starting in youth. There were experiences of unpleasant or abusive interaction in the family environment. Participants had few memories of pleasure, and, if they did, these tended to be related to achieving or performing something with the body. Typical initial BDI scores were 26 or more, and there was no, or very little, change in the BDI score post-intervention. The body image quality also tended not to change, or even got more difficult.

*Moderate case* participants were characterized by the situation, that three of the four variables of activity level, body image quality, relationship style, and initial BDI were in the difficult range. Difficult range meant that the relationship style was insecure, the activity level was seldom good, the BDI score was on the higher level, typically 18–22, and the body image quality was neutral or on the negative side. There was ambivalence and flux in how the participant experienced their body. There could be better moments, but dislike, shame, and a sense of their body (and themselves) not being sufficiently good tended to dominate the body image. Body activity could be troubled by pain or worry, or the participant had a sense of having moved more in the past. There were memories of disruptive interactions in various environments, whether with abusive parents, school mates, or superiors. Post-intervention BDI score was typically 16, which had lowered a few points from pre-intervention level and indicated mild depression. The body image quality might change slightly for positive, but it was typical that the ambivalent, fluxing quality continued to remain after a short intervention.

*Potential for full recovery* participants had a safe attachment style and initially quite a good activity level. Their body image quality was neutral or moderately low, but they expressed a basic positivity about their bodies and body activity. This type of participant connected positively with body action and could safely tolerate discomfort in embodied activities – for example, the rigors of work or outdoor activities – and still consider the activity good. Participants expressed thoughts about, and experiences of relating to others when describing their embodied experiences. The relationships may have had troubles and tensions, and participants named these. However, they were also able to note and relate to positive relational experiences with others. Initial BDI scores were typically 20 or less. Post-intervention BDI score was below 10, i.e., participants had normal mood.

A small group of participants initially had high BDI scores (range 16–39, mean 27 points), negative body image quality, and low activity level. Their relationship style tended to be insecure, i.e., fearful/disorganized or preoccupied/contradictory. These participants were older than the mean age of 42 – their mean age was 48. Despite their difficult pre-intervention situation, these participants benefitted greatly from the treatment. They were named “*mystery cases.*” Their post-intervention BDI scores ranged between 8 and 17, with a mean of 12 points, indicating minimal symptoms. Some of these participants did not remain at this level, thus the 3-month follow-up BDI score mean increased to 16 points. This, however, was still a notable improvement over their initial scores. Pre-intervention BIA scores were negative for 88% of this subgroup. Post-intervention BIA scores showed some changes: while 57% of scores remained negative, 43% had shifted to moderately positive scores. However, changes in BIA score were not a clear indicator for the larger changes in BDI scores. Analysis of the feedback these participants had given showed that for them the group experience, while not necessarily easy, was clearly meaningful and positive. Participants felt thankful for the experience and considered it important to them. This particular subgroup elicits an assumption that the group experience was a factor that may have had a strong impact on the amount of change in some participants’ scores. Yet it is impossible to predict in advance, how a group process will emerge in a group, which is beginning its process.

As to how these four types of cases fitted into the two participant body image profiles generated by LPA, the difficult cases, mystery cases, and those moderate cases whose BIA score change was low were in the negative body image profile. The moderate cases whose initial BIA score was neutral, or who experienced greater change in their BIA scores, and the potential for full recovery cases were in the neutral BIA profile.

## Discussion

The main goal of our research was to investigate the contents of body image among participants with depression. The body image of the participants was characterized by low energy and low activity.

The relationship with own body is troubled: there is often shame and disgust toward own body. The body does not feel good, the connectedness with body sensations is weak and uncomfortable. Breathing is frequently not free. There is much embodied concern in social situations, which is experienced in tension and anxiety in the body, and in ruminative thinking about how others perceive one’s body and actions. Moments alone may be loaded with fear and restlessness, though often participants reported they felt more relaxed when alone than with others. There is variety to this uncomfortable and troubled body image, as some participants were aware of the changing quality in how they sense and feel about their embodiment. Also, some participants initially reported some positive experiences in relation to their body: liking physical activity, accepting one’s body and finding a sense of connectedness with one-self through body and physical activity. These findings are similar to the earlier body image findings in a smaller sample by Pylvänäinen and Lappalainen (2018): low energy level, lack of the sense of agency in social situations, shallow experience and tense habitation of their body.

The DMT group intervention resulted in change in body image. The change could be observed in the more accepting quality of body image and increased expressions of options in relating with the body. Two participant profiles were detected in the data with the LPA, namely a participant with negative body image and participant with neutral body image profile. For a participant with negative body image profile, there was minor change in the body image quality, and some change in mood. For the participant with neutral body image profile, there was more change for positive in the body image quality and mood improved. A secure attachment style was the most apparent style for 30% of the participants in the neutral body image profile, while for the participants with negative body image profile only 15% considered their attachment style is secure. Also, neutral body image participants reported better mindfulness skills than the negative body image participants. The differing factors were acting aware, non-judgmentality and non-reactivity.

The body image quality is significant factor in depression and in recovering from it, but essentially, the phenomena are systemic. The relevant interplaying factors are body image, attachment style, activity level, the severity of depression and mindfulness skills. In addition, sometimes even with a complicated initial condition, the experience of the creative DMT group process can produce change that is more positive that could be expected on the basis of the difficult starting condition. Those patients who recovered into normal mood during the short-term DMT group intervention, typically initially had neutral body image, had a secure attachment style, had fairly good activity level and reported moderate depression level.

### Clinical Implications

Body Image Assessment was used in this study to collect information about the initial condition of participants, and to describe the changes that occurred during the research period. Both 45- to 60-min face-to-face BIA interviews and written BIA responses yielded similar themes. In therapy practice, the BIA interview is a way to build a therapeutic alliance between the participant and the therapist. Inquiry about the participants’ embodied experiences can be a new experience for them, and, when approached in a non-judgmental manner, can be a validating moment in which participants feel heard. Attention is given to embodiment – it is no longer ignored.

Body Image Assessment data provided a relevant perspective on the bodily experiences that patients with depression have had. This is very informative for DMT practice, but this perspective is helpful in general psychiatric treatment as well. It can assist in recognizing appropriate approaches to treatment and goals that would help the patients to connect better with themselves through increased awareness of, and connection to, their body in a revitalized way. These changes are essential to proper recovery from depression.

### Thoughts on Body Relationship in Cultural Context

Dance movement therapy has its roots in dance as an art form. In the Western culture, DMT developed from the observation that expressing oneself through dance and engaging in shared dance experiences can have beneficial effects on the sense of self, self-actualization, and vitality (Chaiklin, 2009). As dancers increase in skill, they must develop sensory attentiveness in the observation of kinesthesia in themselves and in co-dancers. The dancer needs physical strength and co-ordination of bodily movement, and, in order to put these to skillful use, must sense what is happening in her body, how the body functions and what it is the feeling (see for example Bartenieff, 1980; Buckwalter, 2010; Fernandes, 2015). Our data on the body image of the patients with depression showed, this attentive connection to the body, which is a necessity for the dancer, was not a habit among the research participants. The body memories expressed traumatization many of the patients with depression have suffered, and traumatization may be one factor contributing to the poor connection with the body sensations and the wish, or practical habit of avoiding them.

It is relevant to remember, that body image and the way we relate to our body is shaped by our interactions with others and by the cultural habits we maintain in relation to the body. One element of body image is body memory, and in the taxonomy proposed by Fuchs (2012) there are several forms of body memory that are specifically shaped in interaction with others: inter-corporeal memory, which is related to the most basic, pre-thematic and bodily contact with other subjects; incorporative memory, which contains the bodily imitation and identification with others, and social roles that shape one’s embodied expression; situational memory of affectively charged lived situations; and traumatic memory. The body image is not just our own making – it is shaped in interactions with others (see also Summa et al., 2012).

In the 18th and 19th centuries, sociologists and philosophers such as Hegel, Weber, and Durkheim addressed the phenomenon of alienation (see Schabracq and Cooper, 2003; Rae, 2012). Alienation refers to a sense of disconnectedness, feeling estranged from surrounding society because of social situations of labor, living circumstances, and modernization. Later, Seeman (1959) proposed five prominent features of alienation: powerlessness, meaninglessness, normlessness, isolation, and self-estrangement. The current urban culture evolves more and more around media, applications, and virtual reality. Consequently increasingly less complexity in motor actions is needed, or even allowed, in daily life. Alienation is evolving to the next level: the individual is becoming alienated from their own body, and the bodies of others. The questions of body-connectedness or embodiment have not been addressed in the discussion of alienation, but in our data on the body image of the patients with depression, one can observe a resonance of similarities in their body image contents and the described qualities of alienation. A question emerges: do the current cultural habits of treating the body and embodied relating to one another enforce the alienation and sensory disconnection with the body? Bringing more awareness and interest to this layer in the culture, might open new ways to create resiliency among people against depression and to support the recovery from it.

### Study Limitations and Future Research Ideas

This study focused on the body image of the patients with depression and explored the association between embodied experiences and depression level of the participants. The data collection relied on self-assessment tools and on verbal interview/inquiry. This creates a limitation for our data, as at the core there is a translation from sensory and lived experiences into verbal expression. The sensitivity of the word-based self-evaluation tools may be impaired by the participants’ difficulty in identifying their own characteristics among the sentences and descriptions. The scoring system of the BIA is very simplified and insensitive to discrete changes. Positively, this may assist in perceiving general trends in the data but may also result in poor detection of more subtle changes.

In clinical practice there may be a need for quick initial screening of body image. It would be helpful, if this could be done with a questionnaire that does not need a long time to respond nor to analyze, and the use of the screening tool would not need much training for the staff. A selection of sentences with a Likert-type scale for rating them would be practical. For research purposes, the development of this quick assessment tool would enable and make more efficient the study of larger research samples.

Thus far in research, BIA has been used to capture how depressed patients in psychiatric treatment and in DMT groups perceive their body image. BIA could and should be used to gain more information about the body image of people who consider themselves high-functioning and in health. This would aid in understanding, what factors could contribute to developing resilience, supporting flexibility, give a sense of agency in action, and shape an integrated body image. The current assumption is, that the central factor in these is the sufficient sense of safety in interactions the person has experienced in both their past and present (Siegel, 1999; Porges, 2009; Pylvänäinen, 2018). Knowing more about these factors and their systemic interactions would be helpful the prevention and treatment of depression.

## Data Availability Statement

The raw data supporting the conclusions of this article will be made available by the authors, without undue reservation.

## Ethics Statement

The studies involving human participants were reviewed and approved by the board of research ethics in the Central Finland health care district (Dnro 8U/2016). The patients/participants provided their written informed consent to participate in this study.

## Author Contributions

PP was responsible for conceptualizing this manuscript, its organization, drafting, and finalization of the manuscript. PP also conducted qualitative analysis of the body image data with Atlas.ti. KH was co-researcher in the larger study, contributed to the description of the LPA analysis and offered valuable comments on the manuscript. JM was responsible for LPA and other statistical analysis of the data. JM guided the statistical interpretation of the analysis results. All authors contributed to the article and approved the submitted version.

## Conflict of Interest

The authors declare that the research was conducted in the absence of any commercial or financial relationships that could be construed as a potential conflict of interest.
